# Supplementary material for: A novel, magnetic bead‐based extraction method for the isolation of antimicrobial resistance genes with a case study in river water in Malawi
Source: J Appl Microbiol. 2022 Aug 23;133(5):3191–200. doi: 10.1111/jam.15755 (PMC9804433; doi:10.1111/jam.15755)
Supplement: Supplementary file 1 — Figure S1 [file JAM-133-3191-s001.docx]

**Supporting information headings**

Figure S1: Prepare Homemade sera-mag SpeedBeads Mix sera-mag SpeedBeads and transfer 1ml to a 1.5ml centrifuge tube.

1. Place Speed Beads on magnetic rack until beads are separated.
2. Remove supernatant.
3. Add 1ml TE (pH 7.5-8.0) to beads, remove from magnet, mix by pipetting up and down return to magnet.
4. Remove supernatant.
5. Repeat steps 4 & 5.
6. Add 1ml TE (pH 7.5-8.0) to beads, remove from magnet, mix by pipetting up and down, but DO NOT return to magnet.
7. Add 9g PEG-8000 to a new 50ml conical tube.
8. Add 2.92g NaCl to conical.
9. Add 500ul 1M Tris-HCl to conical.
10. Add 100ul 0.5M EDTA to conical.
11. Fill conical to 49ml using ddH20.
12. Mix conical for 5 minutes until PEG goes into solution.
13. Add 25ul Tween 20 to conical and mix.
14. Add SpeedBeads and TE solution from step 7 to conical and mix.

Fill conical to 50ml mark with ddH20
